# The Institutional Library

**Published:** 1918-09-07

**Authors:** 


### THE INSTITUTIONAL LIBRARY.

### A DEFINITELY-ORDERED SCHEME OF DIETS.

As Sir Robert Burnet suggests, it is doubtless quite true that many medical practitioners cultivate the general rather than the particular when prescribing the form of diet which they judge to be suitable for their patients. It is too readily forgotten that in practice even details which are unimportant in themselves need definition in order to remove doubt and uncertainty and the risk of an agitating domestic controversy. Neither patients nor friends really know sufficient to enable them to distinguish between the essential and the non-essential, and the practitioner ought therefore to supply rules which cover every aspect of his scheme of treatment. The moral effect is always helpful, and, of course, in some respects the material values, too, require recognition. Hence a definitely ordered scheme of diet in many cases of illness is an obligation which the practitioner cannot escape. Sir Robert Burnet's book* is offered as an aid to this duty, and to its efficiency stands the convincing testimony of five editions. The proposals are based on the author's personal clinical experience, and the subject is hardly one for theoretical argument or discussion. We are supplied with an actual list of foods and the hours of their administration for various forms of disease, and the several schemes are in no wise lacking in precision. Necessarily there is a great deal of repetition, and the book is one rather for reference than for continuous reading. But its helpfulness is beyond question, even for those who view the several proposals in a somewhat less serious spirit than that manifest in its pages. If it cannot be regarded as inspiring, it certainly provides sensible counsel and inculcates a useful habit. To call it somewhat old-fashioned is not to suggest a reproach, though it might with advantage, we think, be quickened by an infusion of the modern spirit. Thus in discussing the question of the dietetic treatment of diabetes the book contains no reference to the Allen methods, and, valuable and interesting as were the late Sir Wm. Roberts' investigations on the physiology of digestion, these can hardly be described as "recent" in the sense in which this word is used in scientific writings.

* *Foods and Dietaries. A Manual of Clinical Dietetics.* By Sir R. W. Burnet, K.C.V.O., M.D. Fifth Edition. (London: Charles Griffin and Company, Limited, 1917. Pp. 204 + xii. Price 4s. net.)

### THE TWENTIETH EDITION OF A GREAT WORK.

The present edition of this well-known text-book* appears exactly sixty years after Henry Gray completed his original volume. Gray was then thirty-one years of age, and held the position of lecturer on anatomy at St. George's Hospital. He had already an established position in the scientific world, for he was elected a Fellow of the Royal Society some years previously and had been awarded the Astley Cooper prize of three hundred guineas for a dissertation on "The Structure and Use of the Spleen." In 1860 a second edition of the "Anatomy" appeared under Gray's own direction, but this was unhappily his last contribution, for he died in the following year of confluent small-pox. A career of great promise was thus prematurely closed, and one cannot fail to sympathise with the pious determination to continue to associate the name of the original author with the later editions of his work. St. George's Hospital medical school rightly numbers Gray's work among its honoured traditions, and some of the best known of its teachers have taken an active share in the presentation of the several issues that have been required. Thus the editions from the third to the ninth, inclusive, were edited by Timothy Holmes, and the tenth to the fourteenth, inclusive, by T. Pickering Pick. In the fifteenth and sixteenth editions Professor Howden collaborated with Mr. Pickering Pick, and in the last four editions he has been the sole editor. The association of the volume with St. George's Hospital is maintained in the present edition by the inclusion of "Notes on Applied Anatomy" by Dr. A. J. Jex-Blake and Mr. Fedde Fedden, members, respectively, of the medical and surgical staffs of the hospital.

A problem for every writer on systematic anatomy nowadays is whether to adopt or to ignore the "Basle" terminology. The early enthusiasm for the uniform nomenclature has been somewhat clouded in recent years, and not a few authorities in this country have set them selves definitely against it. The person most to be pitied in these divided counsels is the unfortunate student, who must either become an adept in the use of both the old and the new terms, or must run the risk of meeting an examiner who insists on the terms to which the student has not accustomed himself. Doubtless an examiner ought to be equal to either event; but even among these select souls the temper is not invariably and inflexibly judicial. Professor Howden, as in his two previous editions, adopts the "Basle" terms, but where the new name differs materially from the old one both appear in the text and all are noted in the index.

There is no difficulty in accounting for the success of "Gray." First may be mentioned the excellence and teaching value of the illustrations. This, it appears, was a feature of the first edition, the drawings for which were made by Dr. H. Vandyke Carter, a demonstrator of anatomy at St. George's Hospital. The tradition has been well maintained, and it is not too much to say that there is no other text-book in English which is so well provided with diagrams and drawings that are really helpful to the student. It is sometimes said that the drawings have a diagrammatic quality, and that to this extent they depart from strict fidelity to Nature. But even if this proposition be allowed it is not less true that what is here exhibited is a most effective representation of facts, and that the student will learn from the pictures what it is essential he should know and should understand. A great

* *Anatomy Descriptive and Applied.* By Henry Gray, F.R.S., F.R.C.S. Twentieth Edition. Edited by Robert Howden, M.A., D.Sc., M.B. (London: Longmans, Green and Co. 1918. Pp. 1324 +xvi. Price 37s. 6d. net.)

**The Institutional Library**—*(continued)*.

help towards this end is the introduction of colour into the illustrations and the naming of the various features of each illustration by marginal references. If the object of illustrations is to teach through the eye and to impress the visual memory the scheme adopted in this manual of anatomy needs no defence. We desire to give to it an expression of high appreciation, and in so saying we believe we express the feeling of medical students in all the schools of this country.

A second reason for the success of this book we find in the consistent fashion in which the essential purpose of the volume governs its construction. Its ambition is to teach human anatomy to students who have for their main aim the practice of the medical and surgical art. Anatomy may doubtless be studied for other purposes, and may even be an end in itself—a mental discipline, a philosophical inquiry, a refreshing adventure. Now we are far from saying that the medical student is to be preserved from these educational influences. Our point is that they are secondary or subordinate, and that the essential thing for the future practitioner is the acquisition of such a knowledge of anatomy as is necessary to sound medical and surgical practice. With a good foundation soundly laid the building may be carried to any desired height, and it is because "Gray" provides this firm foundation that the book is commended by teachers and adopted by students. There are, we know, more pretentious volumes, and we have no desire to minimise their value. But we must confess that they do at times seem more anxious to display erudition than to supply help to the student. Whatever else anatomy may be, it is certain that in the medical curriculum its main end is to contribute a basis of knowledge essential to medical and surgical practice. In the volume now before us this aim is steadily kept in view, and in this respect, as in others, Professor Howden has proved himself an admirable editor. The call for four editions in the nine years during which he has exercised this office is a fact which speaks in no uncertain tongue. We cordially congratulate him on his achievement, and we doubt not that he has many more successes to attain. The abundance, the distinctness, and the teaching efficiency of the illustrations, the lucidity and practical qualities of the text—these are the distinctive characters of Gray's "Anatomy," and they are fully preserved and emphasised in this, the twentieth, edition.

**Medical Contributions to the Study of Evolution.** By J. G. ADAMI, M.D., F.R.S. Pp. xi.+372. Large octavo. (London: Duckworth and Co. 1918.)

IN a paper which is called THE HOSPITAL and not *The Laboratory* one could hardly argue that medicine should be predominantly a science, instead of, as at present, predominantly an art. And no doubt such a change would be viewed with regret by many people. Nevertheless, the trend of events points that way, while the cause of progress and enlightenment dictates the warmest of welcomes to any notable scientific contribution from one of our calling. Now, unfortunately, although Dr. Adami is a distinguished man, one cannot call his present contributions notable. So much should be said at once, for whatever may be thought of Voltaire's advice to epitaph writers, nobody can deny that it is binding upon reviewers. In the first place, this volume is not a homogeneous work, but a collection of papers written or read at previous dates; and the bearing of some of these papers on evolution, large subject though that is, is but fragmentary, so that there is a great deal of irrelevant matter to get through. Had Professor Adami lopped off the protruding heads and tails of his chapters, selected amongst their members, and then wrought and digested what he had before him into an ordered and congruous whole, it would have been more trouble to him, but less to us, and a good deal less to the printer. We might have had two hundred striking pages, conveying well the main theme, which is the permanent modification of low forms of life by means of artificial environment; a principle classically illustrated forty years ago by the experiments of the biologist Dallinger. Of course, bacteriology and pathology can be drawn on for many examples of the fact in question, and Dr. Adami, an accomplished pathologist of ripe experience and wide knowledge of his subject, puts forward very many. But in so doing, he, like some other medical men, indulges in invective of a fustian order which dismays, not those at whom it is flung, but rather those whose sense of solidarity makes them anxious for the credit of their profession in the great world of learning. If only as a matter of tactics, the controversial Appendix II. should have been omitted—and this opinion is offered in full remembrance of the old Persian's saying that science and controversy are almost inseparable.

**Chemistry for Beginners.** By C. T. KINGZETT, F.I.C., F.C.S. (London: Baillière, Tindall, and Cox. Third Edition. 2s. 6d.)

WE welcome the appearance of the third edition of this little book. It is at once clear, concise, and comprehensive, and affords an admirable introduction to the basal principles of chemistry. The boy who masters it will have the root of the matter in him. We are pleased to see the author has added a new chapter "which can be used by teachers and students alike for making practical acquaintance with the subject." We must beware of training the rising generation as mere theorists. The future of the nations, in a material sense, would appear to be very largely in the laboratory, and if we are to maintain our position it will not suffice that we give those who are to come after us, and who will enter into the scientific competition that lies ahead, a merely academic knowledge. They must be chemists, and a man without practical acquaintance with retort and crucible can no more be a chemist than a man who has no practical experience with a stethoscope can be a physician.

**Transactions of the Incorporated Sanitary Association of Scotland, 1917.**

THIS volume includes a number of excellent papers and business-like discussions, and it records on various topics the views of medical practitioners, medical officers of health, veterinary surgeons, sanitary inspectors, and the representatives of local health authorities. To judge from the list of delegates and speakers, the Association would seem to be widely representative of Scottish opinions. As illustrating the subjects discussed we may mention "Housing of the Working Classes," "Rates and Taxes on House Property," "Ratepayers their own Landlords," "Qualification for Sanitary Inspectors," and "Sterilisation of Tuberculous Flesh." The address by the President, Mr. F. G. Holmes, C.E., on "The Practical Application of Modern Sanitation" is a very attractive contribution to social science and the art of life. Topics of the hour are well represented, as by a discussion "Is a Ministry of Health Desirable?" and a paper on "The Settlement of our Sailors and Soldiers in Rural Occupations after the War." Another practical item is an account of a popular lecture and cooking demonstration on the use of "War Flour" by Mrs. Elizabeth L. Waldie, Head Teacher of Cookery in the Glasgow and West of Scotland College of Domestic Science. The Transactions, indeed, are rich in interesting matter and practical suggestions, and they afford an ample justification of the existence of the Association and of the mission which it has undertaken.

